# Separate and concurrent symbolic predictions of sound features are processed differently

**DOI:** 10.3389/fpsyg.2014.01295

**Published:** 2014-11-18

**Authors:** Marika Pieszek, Erich Schröger, Andreas Widmann

**Affiliations:** Cognitive incl. Biological Psychology, Institute of Psychology, University of LeipzigLeipzig, Germany

**Keywords:** prediction, ERPs, auditory processing, hierarchical processing, audiovisual, symbolic, IR, N2b

## Abstract

The studies investigated the impact of predictive visual information about the pitch and location of a forthcoming sound on the sound processing. In Symbol-to-Sound matching paradigms, symbols induced predictions of particular sounds. The brain's error signals (IR and N2b components of the event-related potential) were measured in response to occasional violations of the prediction, i.e., when a sound was incongruent to the corresponding symbol. IR and N2b index the detection of prediction violations at different levels, IR at a sensory and N2b at a cognitive level. Participants evaluated the congruency between prediction and actual sound by button press. When the prediction referred to only the pitch or only the location feature (Experiment 1), the violation of each feature elicited IR and N2b. The IRs to pitch and location violations revealed differences in the in time course and topography, suggesting that they were generated in feature-specific sensory areas. When the prediction referred to both features concurrently (Experiment 2), that is, the symbol predicted the sound's pitch and location, either one or both predictions were violated. Unexpectedly, no significant effects in the IR range were obtained. However, N2b was elicited in response to all violations. N2b in response to concurrent violations of pitch and location had a shorter latency. We conclude that associative predictions can be established by arbitrary rule-based symbols and for different sound features, and that concurrent violations are processed in parallel. In complex situations as in Experiment 2, capacity limitations appear to affect processing in a hierarchical manner. While predictions were presumably not reliably established at sensory levels (absence of IR), they were established at more cognitive levels, where sounds are represented categorially (presence of N2b).

## Experiment 1: separate predictions

### Introduction

Human perception is currently understood as a complex and active process. This mainly relates to the fact that the processing of incoming information is heavily and already early biased by what we have experienced before. The current research investigates underlying mechanisms, involving knowledge stored as internal representations of the environment. The knowledge shapes the structuring of new information to infer its causes and leads to interpretations (perceptual inference); it also predicts outcomes of the currently experienced situation (den Ouden et al., 2012). The predictive principle can improve behavioral adaptation in this situation due to facilitation and speeding-up the processing (Bar, 2007; Bubic et al., 2010; Wacongne et al., 2011; Arnal and Giraud, 2012; Clark, 2013). The internal representations work as generative models, i.e., they predict upcoming events on basis of experienced rules of the environment (Bar, 2007; Winkler et al., 2009; Bendixen et al., 2012; Clark, 2013). The predictive coding theory assumes that predictions of future events are sent to hierarchically lower levels to be matched with ascending information. Resulting prediction errors signal failed predictions. They are forwarded to the higher level to update the generative models for more accurate predictions (Mumford, 1992; Winkler et al., 1996; Bar, 2007; Friston, 2009; Friston and Kiebel, 2009; Bubic et al., 2010; Wacongne et al., 2011; Arnal and Giraud, 2012; den Ouden et al., 2012; Clark, 2013). Different rules and modalities can be exploited in parallel to reliably predict upcoming events (Horváth et al., 2001; Bubic et al., 2010; Clark, 2013; Schröger et al., 2013).

Visual material can establish predictions for a sound (Bendixen et al., 2012; Lindström et al., 2012; Clark, 2013). Particularly, a mismatch between a predictive note-like symbol and the pitch of the corresponding sound elicits brain responses that signal the violation of a prediction. The Incongruency Response (IR) is characterized as a negative deflection in the difference potential of an incongruent-minus-congruent sound (Widmann et al., 2004; Pieszek et al., 2013). It occurs in the interval of approximately 100–130 ms after sound onset at fronto-lateral sites. The IR presumably reflects the prediction error at sensory levels of processing. At cognitive levels, where the sound is categorized with respect to task affordances (e.g., whether or which button has to be pressed), the N2b is elicited (Widmann et al., 2004; Lindström et al., 2012). The fronto-centrally distributed ERP component is observable at approximately 200 ms after sound onset. N2b in the present context indexes the target detection as known from deviant targets in active oddball paradigms (Ritter et al., 1979; Näätänen et al., 1982).

Associating visual information with a sound involves a higher-order network of associative, multisensory and working-memory related areas (cf. Tanabe et al., 2005). A widely distributed network is also involved when reactivating such association, as was observed when musicians read score (Schürmann et al., 2002; Wong and Gauthier, 2010; Paraskevopoulos et al., 2012). Thus, a prediction for the sound from a symbol is presumably generated in higher-order areas. It is based on the link between the symbol and the previously associated memory representation of the sound, as proposed in the functional model of Widmann et al. (2007). Presumably, the prediction is fed backward to lower processing levels while matched with the ascending information at different hierarchical levels (cf. Friston, 2009). Widmann et al. (2004, 2007) found evidence that the symbols pre-activate auditory memory representations at sensory level. If there is a mismatch, the resulting prediction error (IR) is fed forward to the subsequent level. According to the predictive coding theory (Bar, 2007; Friston and Kiebel, 2009) and models of the mismatch negativity (MMN) mechanism, the prediction error is forwarded to modulate upcoming predictions (Winkler, 2007; Schröger et al., 2013). Additionally, the prediction error may lead to a more elaborated processing of the stimulus by drawing attention to it (cf. Escera et al., 2000), also in the context of symbolic prediction.

In the present studies, we investigated the functional underpinnings and the specificity of symbolic prediction at different levels of the processing hierarchy. In Experiment 1, each symbol defined a specific feature value of pitch or of location in separate blocks. Occasionally, a sound violated the prediction. The participants were instructed to pre-read five symbols, to match them with the presented sounds and to evaluate the congruency between the predicted and the actual sounds. We hypothesized that the IR and the N2b also signal the violation of a sound's predicted spatial location. In Experiment 2, the symbol mapped to one value of each of the two features (e.g., a sound with high pitch and of left location). We investigated whether the brain generates, maintains and matches predictions for the two sound features concurrently. Previous studies reported that two sensory auditory predictions can be maintained and tested concurrently and independently (Levänen et al., 1993; Schröger, 1995; Pieszek et al., 2013). The processes were not dependent on the involvement of attention, that is, task-relevance. At the cognitive-attentive level (as indexed by the N2b), interactions of the representations occurred for the attended regularities (Schröger, 1995; Pieszek et al., 2013). Therefore, in Experiment 2 we expected independent matching processes between the two symbolic predictions and the actual sound at the sensory level (IR). On the cognitive level (N2b), we expected an advantage of the concurrent violation (Schröger, 1995; Pieszek et al., 2013). This would indicate indirectly a parallel, i.e., concurrent processing of the violations.

### Materials and methods

#### Participants

Participants were informed about the non-invasive study according to the Declaration of Helsinki. Particularly, the procedure, the anonymous handling of data and the opportunity to stop the experiment at any time were emphasized. Participants signed the informed consent according to the Declaration of Helsinki before any procedure started. For specific psychological ethical standards we conformed to the ethical guidelines of The German Psychological Society (“Deutsche Gesellschaft für Psychologie,” DGPs, www.dgps.de/index.php?id=96422). Participants received either course credits (students) or money (6€ per h). Additionally, participants received a financial reward according to their performance. Datasets of 19 healthy participants with normal/corrected-to-normal vision and normal hearing were analyzed anonymously (information about their identity was kept separately). The handedness was assessed by a German short version of the Edinburgh Inventory (Oldfield, 1971). Sixteen participants (2 men; 15 right-handed; mean age: 21.9 years, range 18–29) were included in the analysis. Three participants had to be excluded due to technical problems, excessive alpha activity or accuracy below two standard deviations from the mean accuracy (two standard deviations corresponded to a value of 91.1% of correct responses).

#### Stimulation

**Figure 1:**
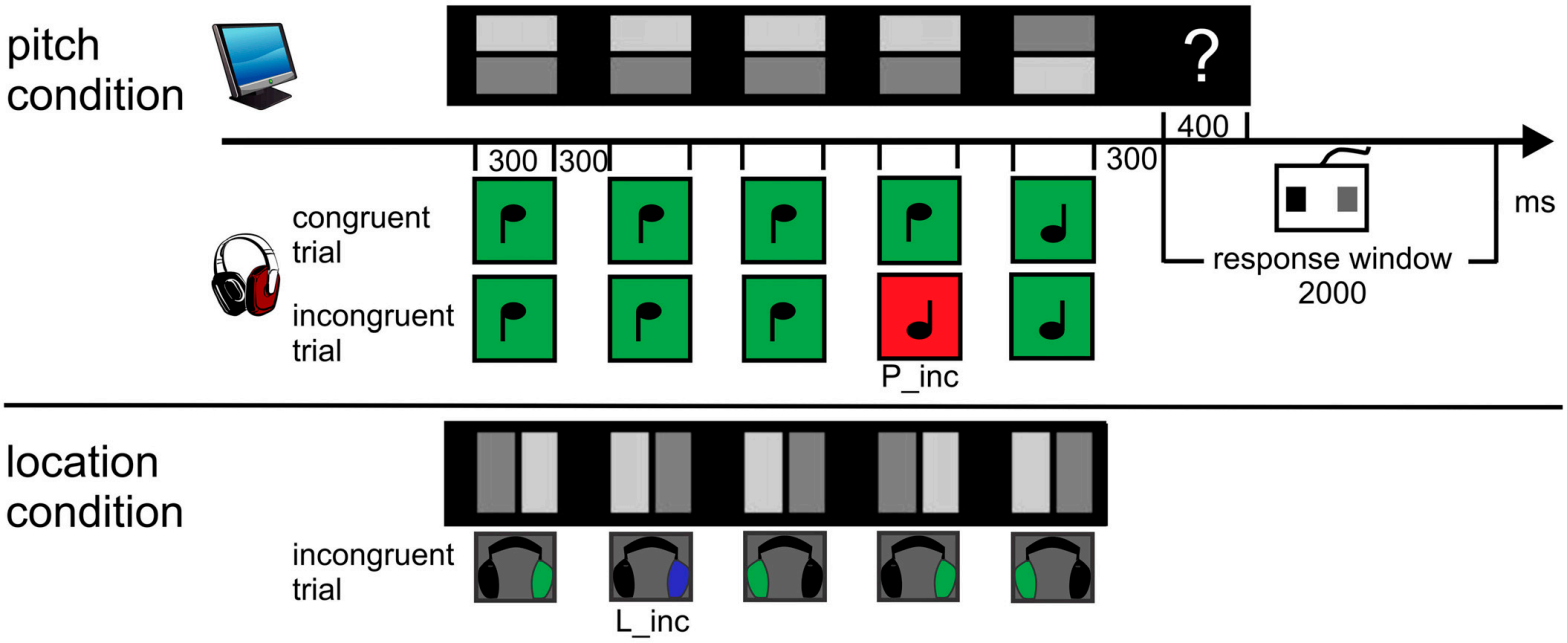
**Procedure and paradigm**. 8 rows consisting of 5 symbols were presented concurrently. The light gray rectangle always predicted the sound feature value. Participants were asked to “pre-read” one row and to match each symbol with its corresponding sound. Following the five-tone melody (one trial), the response cue (“?”) appeared. Participants had to evaluate the congruency of the trial by button press and start to pre-read the next row. The first sound of the next sound sequence closed the response window of 2000 ms. The upper panel displays exemplarily both a congruent and incongruent trial (P_inc at 4th position, red) of the pitch condition. The lower panel displays the example of an incongruent trial (L_inc at 2nd position, blue) of the location condition. Sounds marked as green are congruent to visual symbol.

The visual stimulation consisted of 8 rows of 5 quadratic symbols per block, presented on a CRT-screen 140 cm in front of the participant. It spread over an angle of 4.1 × 9.5°. A trial was defined by one row of symbols with the corresponding sounds, see Figure 1. One symbol subtended a visual angle of 0.5 × 0.5° and consisted of a dark and a light gray rectangle. The light rectangle predicted the value of the task-relevant sound feature. In the pitch condition, it was positioned in the upper or lower half, indicating the high or the low pitch of the sound. In the location condition, it was positioned in the left or right half of the symbol, indicating a sound coming from the left or the right. All symbols persisted on the screen during the auditory stimulation.

The first sound occurred 2000 ms after the onset of the visual display. Sounds arrived binaurally via Sennheiser HD 25-1 headphones. In the pitch condition, two triangle wave tones with the base frequencies of 440 and 352 Hz were synthesized. In the location condition, the tones had a frequency of 396 Hz. The impression of different spatial locations resulted from an interaural time difference (ITD) of 437 μs and an interaural level difference (ILD) of −6 dB between both ears. That is, when the sound was delayed and attenuated on the left ear, participants had the impression that the sound came from the right and vice versa. The duration of sounds was 300 ms (including 10 ms rise and 10 ms fall times). The Stimulus Onset Asynchrony (SOA) of the sounds in each trial was 600 ms. Following each auditory sequence of five sounds (2700 ms) and a subsequent 300 ms-interval of silence, a question mark (Arial, 0.3 × 0.4°) appeared. It was visible for 400 ms at the right side of the corresponding visual row. Its onset defined the start of the response window (2000 ms) which was closed by the onset of the first sound of the following trial. Hence, one trial encompassed 5000 ms to read five symbols, to listen to their corresponding sounds and to respond. One block lasted 47 s. The stimulation was presented with the Cogent Graphics toolbox (developed by John Romaya at the LON at the Wellcome Department of Imaging Neuroscience) via MATLAB R2007b (The MathWorks., Inc.).

#### Design and procedure

The whole session involved about 3 h, the experimental part approximately 47 min. Each of the blocked two conditions consisted of 240 trials, i.e., 30 blocks, providing 1200 sounds. Sounds were randomized within a block. In each condition, 120 trials consisted only of congruent symbol-sound pairs, whereas the other 120 trials contained one incongruent pair embedded in four congruent pairs (= incongruent trial). This resulted in an overall-probability of incongruent pairs, i.e., violations, of 10%. Pitch violations (P_inc) consisted of 50% *high* symbol—*low* sound and 50% *low* symbol—*high* sound. Analogously, location violations (L_inc) were paired *left* symbol—*right* sound and vice versa (see Figure 1). The probability of the incongruent pair in a trial was distributed unequally over the positions (position one and five: each 10%, position two, three, four: each 26.7%). Finally, the number of incongruent pairings per block was randomized within three groups of 80 trials. The order of the conditions was balanced with the response buttons for an incongruent or congruent trial across participants. The first eight participants held the response device horizontally in their hands to press the left (with the left thumb) and right button (with the right thumb). The other eight participants held it vertically to create the impression to press the upper (left thumb) or the lower button (right thumb).

Participants were told how a symbol was associated with the sound. They were instructed to read the symbols of one row in advance and to match each element with its corresponding sound of the five-tone melody. They had to evaluate the congruency of the trial, i.e., to judge whether the predicted features matched the features of the actually presented sounds. The response within the response window should be as correct as possible (= correct response). Before mounting the electrodes, participants trained eight blocks of their first experimental condition. The training of the second condition took place after the first condition was recorded. For each condition, participants had to fulfill the performance criteria (seven correct responses within the response window in two consecutive blocks). This was always the case for the first applied condition, whereas the number of training blocks of the remaining condition (2–8) depended on that. To enforce a high accuracy, participants were rewarded for each correct response with 1 cent in the training (for condition 2 only for 2 blocks) as well as in the experiment. Hence, theoretically they could earn 5.60€ additionally. Actually, participants earned between 5.19 and 5.58€ with an average of 5.43€. After each block, a visual feedback appeared about how much they earned in the block, thus also providing the number of correct responses. Breaks were included on demand.

#### Data recording and analysis

The EEG was recorded with 64 scalp electrodes based on the extended 10-10-system. Additionally, electrodes were fixed at the mastoids, the tip of the nose and according to the triangular layout for the EOG as suggested by Schlögl et al. (2007). The data were analyzed with the EEGLAB open source toolbox for Matlab (Delorme and Makeig, 2004, web: sccn.ucsd.edu/eeglab; The MathWorks., Inc.), statistics were calculated in SPSS 21/22 (IBM). Congruent sounds from incongruent trials and trials with incorrect responses were discarded. The EEG was filtered offline with a 0.1–100 Hz bandpass FIR filter (zero-phase Kaiser windowed sinc FIR, transition band width (TBW) 0.2 Hz, 9275 points, Kaiser beta 5.65 of 0.1% deviation in the passband and −60dB attenuation in the stopband; Widmann and Schröger, 2012). Regression based EOG artifact correction was performed as developed by Schlögl et al. (2007). The continuous data were filtered using a 48 Hz lowpass Kaiser windowed sinc FIR filter (of the same parameters as above, except a TBW of 2 Hz and a length of 931 points). The EEG was segmented into epochs of 600 ms, including a baseline of 100 ms preceding the onset of the sound. Epochs exceeding a threshold of 100 μV at any electrode were rejected. The number of congruent and incongruent sounds was balanced for each participant individually before the grand average was calculated: Congruent sounds were randomly selected as “siblings,” i.e., having the same physical properties and position in the trial as the corresponding incongruent sounds. Epochs were averaged for each participant separately for the two conditions for congruent (P_con or L_con) and incongruent sounds (P_inc or L_inc). The incongruent-minus-congruent-difference waveforms (P = P_inc—P_con; L = L_inc—L_con) and the grand averages of all waveforms were computed.

Latency and amplitude differences between both IR components were tested at Regions Of Interest (ROIs). ROIs were defined separately for each condition (P; L). The mean of four electrodes with the maximum amplitudes in the respective grand-average difference wave determined for pitch ROI frontal (AF3-F5 with bilaterally paired AF4-F6), and for location ROI central (C3-C5 with bilaterally paired C4-C6). Peak latencies of both IRs were extracted with the jackknife method (e.g., Miller et al., 1998) at the respective ROI of each condition within a window of 70–140 ms. It was combined with the relative criterion technique as suggested by Kiesel et al. (2008), using a relative criterion of 100%. Individual latencies were retrieved via the transform by Smulders (2010). The latency means of both conditions were tested with paired *t*-tests for significant differences.

The time window to evaluate amplitude differences was centered at the peak latencies of each IR (pitch: 94–114 ms, location: 113–133 ms). The repeated measures ANOVA was conducted with four factors: feature (pitch vs. location) × congruency (congruent vs. incongruent) × ROI (frontal vs. central) × hemisphere (left vs. right). For each sound feature, a repeated measures ANOVA followed up with the factors congruency (congruent vs. incongruent) × ROI (frontal vs. central). Follow-up paired *t*-tests were performed. Voltage distribution and scalp current density (SCD) maps were computed by using spherical spline interpolation of the scalp potential data (Perrin et al., 1989, 1990). For the estimation of the SCDs, the maximum degree of the Legendre polynomials was 50, the order of splines (m) was 4 and the smoothing factor lambda was 10^−5^.

The peak latencies of the N2b components were determined at Fz with the jackknife method within a window of 100–300 ms, using a relative criterion of 100% (see above). Peak latency mean differences between both conditions were tested with a paired *t*-test at Fz. The paired *t*-test on amplitudes was calculated with the difference potential data from two peak-adjusted time windows (P: 180–220 ms, L: 196–236 ms). Voltage distribution maps were computed by using spherical spline interpolation of the scalp potential data, see above. The reaction times (RTs) and the accuracy data were averaged for each condition for congruent and incongruent trials separately. Please note that RTs cannot be interpreted as participants had to wait for a response cue. The repeated measures ANOVA for the accuracy data included the factors feature (pitch vs. location) and congruency (congruent vs. incongruent). The alpha level was 0.05, two-tailed, for all statistical analysis.

### Results

#### Performance

**Figure 2:**
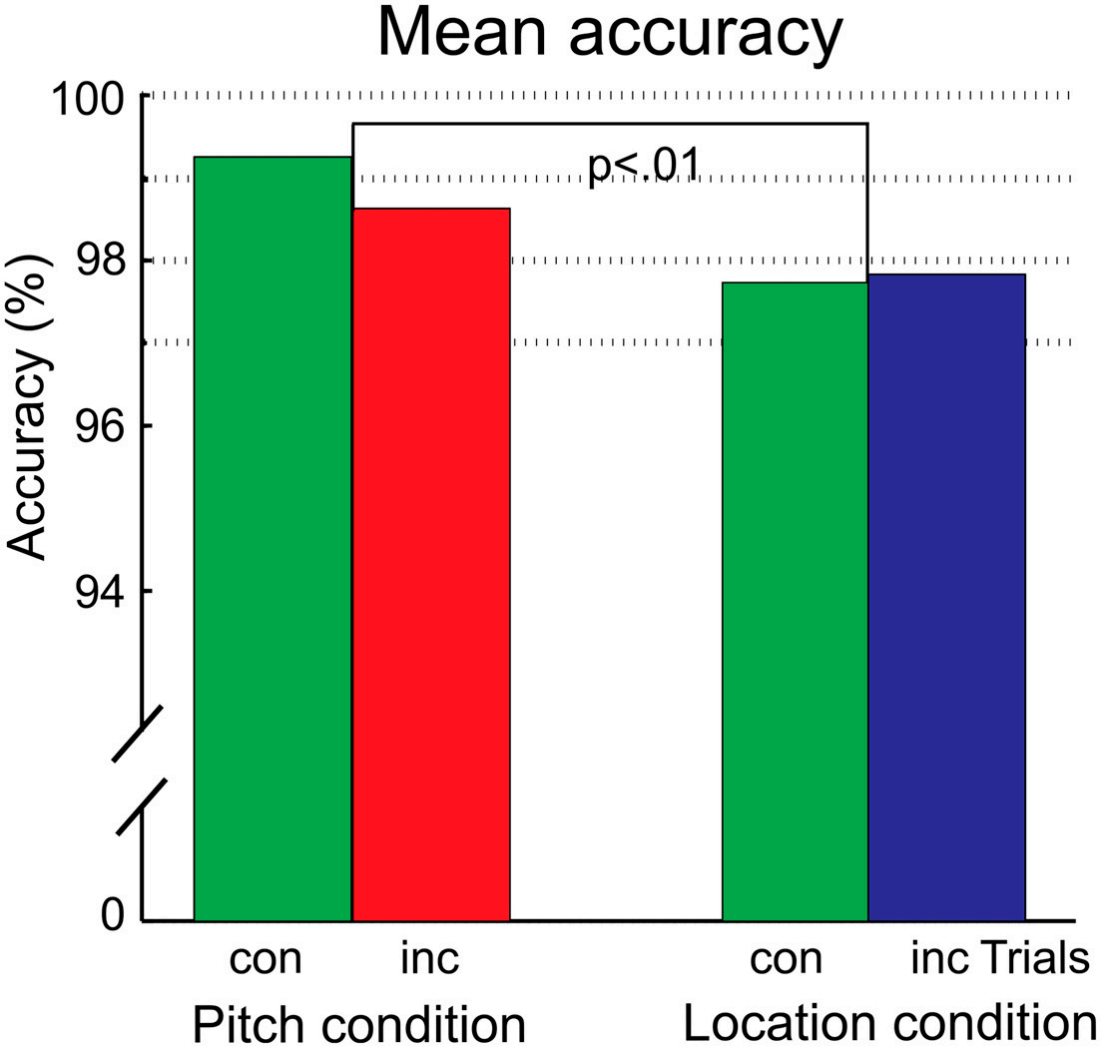
**The mean accuracy for the pitch and location condition, separated for the congruency status of the trial**. The significant difference between the conditions is indicated. There was no difference between congruent (con) and incongruent (inc) trials in any condition.

In the pitch condition, 1.2% of trials were discarded due to responses outside the provided response window. In the location condition, 1.5% of trials were discarded. The results are shown in Figure 2. The accuracy was lower in the location condition than in the pitch condition [feature: *F*_(1, 15)_ = 10.84, *p* = 0.005, η_*p*_^2^ = 0.42]. Accuracy did not differ between congruent and incongruent trials. The RTs were not evaluated statistically due to the use of a response cue. For completeness, the rank order of trials with correct responses is reported. Participants were fastest in pitch trials (congruent: 326 ms, incongruent: 330 ms), followed by congruent trials of the location condition (congruent: 346 ms). The slowest reactions occurred after incongruent trials of the location condition (incongruent: 370 ms).

#### Electrophysiological components of IR and N2b

**Figure 3:**
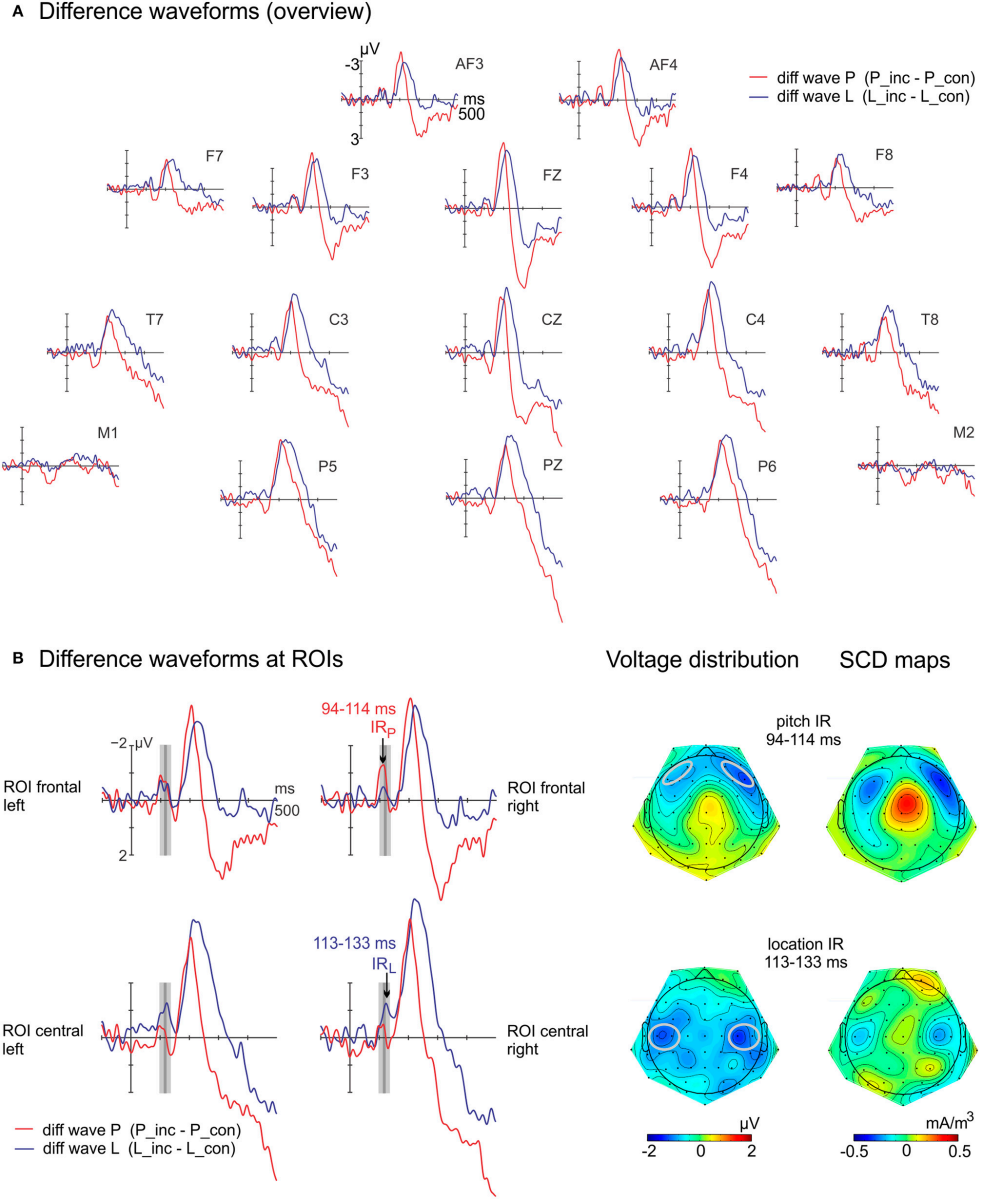
**Difference waves at selected electrodes (A) and at ROIs including IR maps (B)**. (**B** left) The incongruent-minus-congruent difference waves reflect the Incongruency Response (IR) in the marked time ranges for each feature. The plots display the computed ROI frontal and ROI central which are included in the statistical evaluation, separated for hemisphere. (**B** right) Voltage distribution and scalp current density maps (SCD) show different sink-source configurations between the location IR and the pitch IR. The ROIs are marked gray.

The regular auditory ERPs of the two conditions with their respective difference waves (incongruent-minus-congruent sibling) are displayed separately in Figure S1 (Supplementary Material). In Figure 3A, the difference waves of both conditions are displayed at selected electrodes. In Figure 3B left, the two bilateral ROIs used in the statistical analysis of the IRs are shown. Latency differences of the IRs were statistically significant [pitch: 104 ms, location: 123 ms; *t*_(15)_ = 3.1; *p* = 0.008]. The repeated measures ANOVA on ERP amplitudes resulted in the main effects of feature [*F*_(1, 15)_ = 16.7, *p* = 0.001, η_*p*_^2^ = 0.53] and of congruency [*F*_(1, 15)_ = 12.9, *p* = 0.003, η_*p*_^2^ = 0.46]. Further, the interactions of feature × ROI [*F*_(1, 15)_ = 8.0, *p* = 0.013, η_*p*_^2^ = 0.35] and—most importantly—the interaction of feature, congruency and ROI [*F*_(1, 15)_ = 21.2, *p* < 0.001, η_*p*_^2^ = 0.59] were significant. The follow-up ANOVA split for feature showed a significant interaction of congruency and ROI [location: *F*_(1, 15)_ = 8.4, *p* = 0.011, η_*p*_^2^ = 0.36; pitch: *F*_(1, 15)_ = 9.5, *p* = 0.008, η_*p*_^2^ = 0.39]. The topographies of the difference potentials of both features, i.e., the IR components, were significantly different: For location, the difference potential was only significant at the central ROI [*t*_(15)_ = −2.9; *p* = 0.011] but not at the frontal ROI [*t*_(15)_ = −1.4; *p* = 0.192]. For pitch, the *post-hoc t*-tests revealed differences between congruent and incongruent ERP amplitudes at the frontal ROI [*t*_(15)_ = −4.2; *p* = 0.001] but not at the central ROI [*t*_(15)_ = −1.7; *p* = 0.103]. The voltage distribution and SCD maps (Figure 3B right) support these findings. The IR elicited by the incongruent sounds in the location condition reveals a more posterior distribution than in the pitch condition.

**Figure 4:**
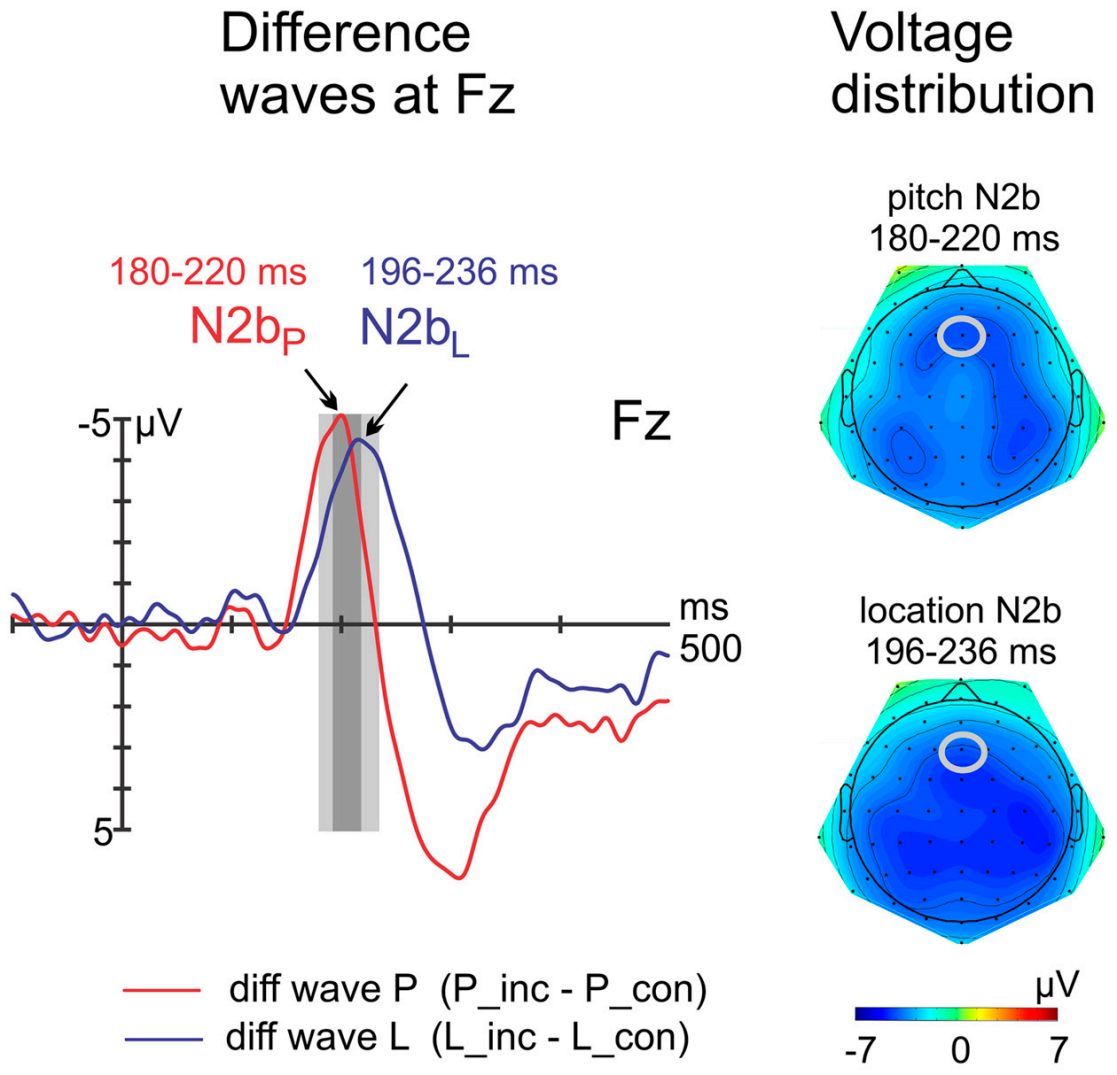
**Difference waves and voltage distribution maps of N2b components**. Left: At Fz, the latency of the pitch N2b peak was shorter compared with the location N2b peak, whereas amplitudes did not differ.

Whereas peak latency differences of the N2b were statistically significant at Fz [pitch: 200 ms, location: 216 ms; *t*_(15)_ = −2.8; *p* = 0.014], amplitude differences computed with separate time windows failed to reach statistical significance [*t*_(15)_ = 0.8; *p* = 0.934], see Figure 4.

### Discussion

We were interested in auditory symbolic predictions for the location of a sound. We compared the IR elicited by violations of location predictions with the reproduced IR for pitch violations (Widmann et al., 2004; Pieszek et al., 2013). In our Symbol-to-Sound matching paradigm, visual symbols and particular sound features are associated via arbitrary rule-based, shortly trained symbols. We assumed on basis of previous evidence that participants are able to actively predict the upcoming sound features. Rare violations occurred and participants had to evaluate the congruency. This approach differs from research investigating score reading by musicians (e.g., Sergent et al., 1992; Schürmann et al., 2002; Brodsky et al., 2003, 2008; Schön and Besson, 2005) which seems a special case of symbolic predictive processes in a particular population. Musical investigations usually involve highly overlearned, long-termed stored knowledge about Western musical rules. The investigated rules rather resemble syntax in language as they stretch over a larger piece of score than note-to-note prediction (cf. Rohrmeier and Koelsch, 2012).

As expected, violations of the prediction for a particular pitch and for a particular location elicited error signals at an early, more sensory level (IR) and at a later, more cognitive level (N2b). The results are in line with the proposed functional model by Widmann et al. (2007) for symbolic prediction processes. Here, a symbol pre-activates associated auditory sensory memory representations. The representations are matched with the incoming pre-analyzed sensory information of the actual sound. A mismatch of the representation elicits the IR and the N2b. The brain responses reflect the detection of the violation and draw attention to the sound in the following (cf. also Widmann et al., 2004). The results also fit to the predictive coding theory which proposes a bidirectional message passing in a hierarchical system (Mumford, 1992; Winkler et al., 1996; Bar, 2007; Friston, 2009; Friston and Kiebel, 2009; Bubic et al., 2010; Wacongne et al., 2011; Arnal and Giraud, 2012; den Ouden et al., 2012; Clark, 2013).

The voltage distribution and SCD maps suggest that both IR components were elicited in auditory areas. Both sort of maps for pitch resembled the ones received from pitch violations in a previous study (Pieszek et al., 2013). Especially the SCD maps contain valuable information. They reduce the spatial smearing of voltage and provide a reference-free view on voltage distributions (Giard et al., 2013). Remarkably, the sinks of the location IR emerged more posteriorly, and the location IR peaked later than the pitch IR. This suggests that (partly) different generators were involved which are presumably located in feature-specific areas of the cortex. Several functional or anatomical unimodal studies corroborate the finding that prediction violations in different sound features are processed independently of each other (Giard et al., 1995; Schröger, 1995; Molholm et al., 2005). Distinct encoding paths of spatial and pitch information (Alain et al., 2001) may account for the observed differences in latency and topography in the present study. Deouell and Bentin (1998) also reported that there exist inherent differences in processing of different sound features with respect to latencies and amplitudes, as indexed by MMN.

Subsequently, N2b components of comparable amplitudes were elicited in both conditions. N2b is usually elicited by task-relevant deviants in oddball paradigms (Ritter et al., 1979; Näätänen et al., 1982; Novak et al., 1990) and in visual-auditory paradigms with a response task (cf. Lindström et al., 2012; Pieszek et al., 2013; but Tervaniemi et al., 2003: no response was required). Functionally, the N2b is assumed to indicate a “decision process related to sensory discrimination of attended stimuli,” i.e., attentive target detection (Ritter et al., 1979, p. 1360). In the present paradigm, targets were defined by the violation of a symbolic rule-based prediction. Hence, the N2b reflects here the detection of such violation at a cognitive level involving attention (Lindström et al., 2012). Remarkably, the peak latency of the location N2b was prolonged relative to pitch. The difference may be transferred from sensory processing as it was approximately of the same amount as the IR latency difference. However, the observed processing delay and the lower accuracy in the location condition suggest an impaired processing compared with the pitch condition. Using headphones may account for this finding. While it does not affect pitch processing, the processing of spatial location information is impaired as the head-related transfer function (HRTF) of the headphones was not adapted to each participant's pinna.

In sum, the prediction violations of both features were detected on both focused levels in the processing hierarchy. The successful detection led to comparable response accuracies as in congruent trials. Hence, the symbolic prediction process can be generalized to some extent but other features need further investigation. Further, the IR results suggest (partially) different processing areas. However, in natural life there is probably more than one prediction violated when new events occur. Experiment 2 was designed to investigate the functional aspects of concurrent prediction violations of two features at the sensory and at the cognitive level.

## Experiment 2: concurrent predictions

### Introduction

In Experiment 2 we sought evidence of two predictive models established intentionally by a single visual symbol. How and at which levels of the processing hierarchy are these predictions maintained and matched to the incoming input? The additive model (e.g., Schröger and Widmann, 1998; Paavilainen et al., 2001; Besle et al., 2005; Sella et al., 2014) can demonstrate how processing takes place. According to the model, the measurements of two single conditions are added and then compared with the concurrently measured condition. As long as the model holds, concurrent processing of the conditions is assumed as independent. Concurrent processing is assumed as interactive, when the sum of conditions results in super- or sub-additivity compared with the concurrently measured condition.

Previous studies on concurrent predictions involved different levels of attention. Results showed that two predictive models can be maintained and tested independently, i.e., separately and concurrently, at sensory levels. In later processing, predictions start to interact. For instance, additivity of the pitch MMN and location MMN showed that the underlying representations and matching processes are independent from one another at the sensory level (cf. Levänen et al., 1993; Schröger, 1995). Schröger (1995) used a passive-listening (sounds are not task-relevant) and an attentive (sounds are task-relevant) auditory oddball paradigm. As expected, N2b components were only observed when sounds were task-relevant. The subadditivity at the cognitive level, as indexed by N2b, suggested an interaction of the activations by the single violations. In another study, a symbol (within the trial) and the ongoing auditory stimulation (across trials) induced concurrent pitch predictions (Pieszek et al., 2013). The intentional symbolic prediction was useful for the auditory discrimination task, whereas the auditory regularity (oddball paradigm) did not have any task-relevance. Hence, the captured regularities (and thus the predictions) originated from different sources and involved different levels of attention. The additive model showed that both predictions of pitch co-existed independently at the sensory level (as indexed by the IR and the MMN) whereas they interacted at the attentive level (as indexed by the N2b). At the sensory level, even contradictory predictions for the upcoming sound's pitch were maintained and tested in parallel due to the modularity of the matching mechanisms.

The present study investigates concurrent symbolic intentional predictions of a sound's pitch and location. As far as possible, the same design, settings and processing steps of the data were applied as in Experiment 1. We assumed that the IR may indicate the detection of every violation type at sensory levels whereas the N2b may indicate the attentive detection. Referring to Experiment 1, we expected to find a temporal advantage of the processing of the pitch violation compared with the location violation. Amplitude differences between pitch and location violations were not expected. Moreover, we hypothesized that the concurrent violation would show a processing advantage compared with the single violations. This could be shown by a higher amplitude for the concurrent violations (Schröger, 1995; Pieszek et al., 2013). Alternatively, we hypothesized that establishing, maintaining or testing of predictions may be impaired for either one or for both features. For instance, the task might be too demanding to encode, maintain or test concurrently two features.

### Materials and methods

#### Participants

Participants fulfilled the same criteria and procedures as described in Experiment 1. Data from 21 healthy participants were recorded of which none took part in Experiment 1. The data of five participants were discarded (four did not fulfill the criterion of 90% of valid responses, another reported non-conformity with the instruction). Sixteen participants (3 men; 15 right-handed; mean age: 25.4 years, range 20–33) were included in the analysis.

#### Stimulation

**Figure 5:**
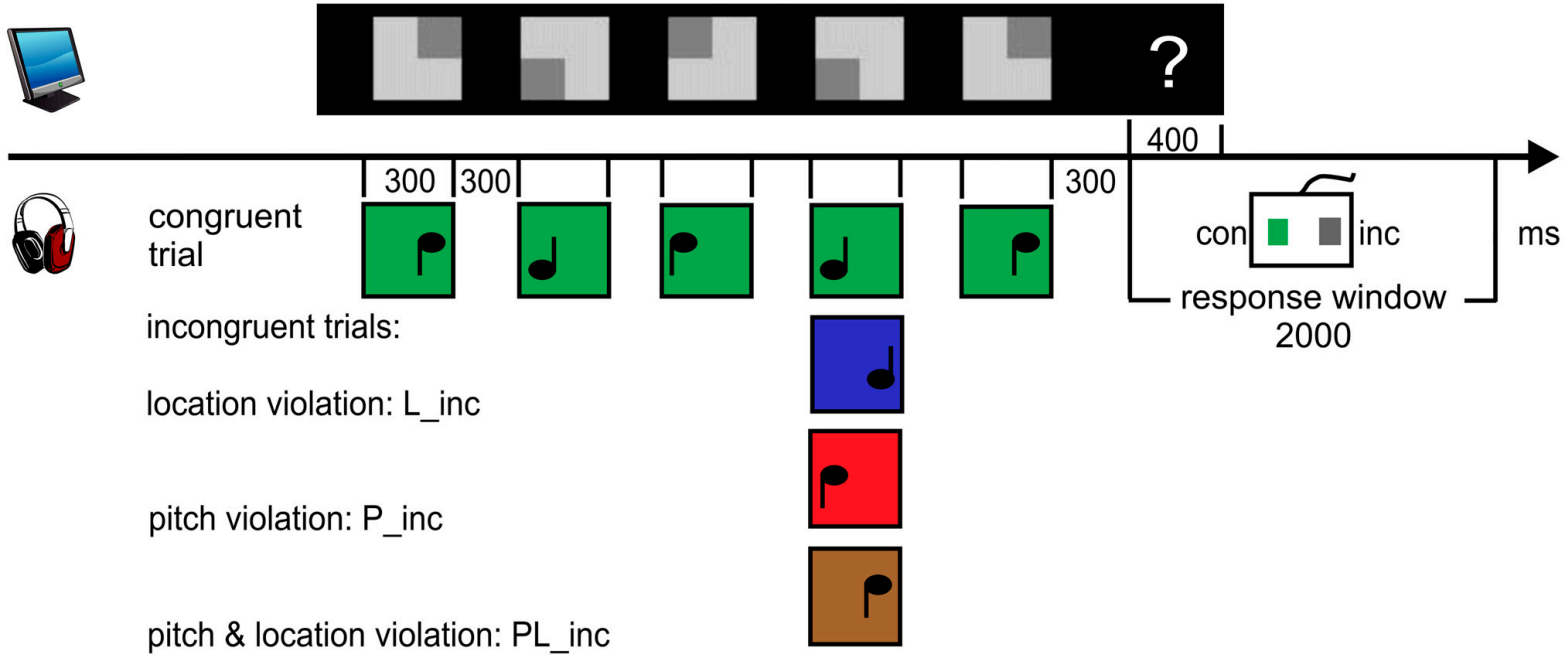
**Procedure and paradigm**. Within a row (trial), the elevation of the dark corner was roughly comparable to a musical score with respect to the pitch prediction (high or low). Its lateralization corresponded to the lateralization of the sound (left or right). In the incongruent trials, one out of the five sounds violated the prediction of either location or pitch or both features at the same time. In total there were 10% violations, shown here exemplarily at position 4. In the experiment, incongruent pairs were balanced for their position in the trial. Participants (*n* = 16) were asked to “pre-read” the symbols. After each trial (“?”), they were asked to press the right button when a sound violated the prediction and the left when the sounds were all congruent. 2000 ms after onset of the question mark, the sound sequence related to the next visual row (8 per block) started. The violations are color-coded.

The stimulation parameters were kept exactly the same as in Experiment 1 except that symbols and sounds were modified, see Figure 5. The symbols were designed to allow for equal discriminability of pitch and location information, whereby the dark corner symbolized the sound feature. The upper-left corner predicted the high sound coming from the left and the lower-left corner predicted the low sound from the left. The upper-right corner predicted the high sound from the right and the lower-right corner the low sound from the right. The equiprobable triangle wave tones had base frequencies of 440 or 352 Hz and an intensity of 70 dB SPL. Each sound was binaurally presented but gave the impression of coming from left or right (cf. “Materials and Methods” of Experiment 1: ITD 437 μs, ILD −6 dB).

#### Design and procedure

The whole session lasted about 4 h with an experimental time of approximately 70 min. In total, we applied 90 blocks (3600 sounds) consisting of 720 trials. One block consisted of 8 intermixed trials of 0:47 min duration. Half of the trials (360) contained only congruent pairs (Con), the other 360 trials contained one incongruent pair among four congruent. Thus, 10% violations in total resulted in 120 pitch violations (P_inc), 120 location violations (L_inc) and 120 concurrent violations (PL_inc). Each violation type contained the same percentage (25%) of the four possible incongruent pairings, e.g., for pitch: *high*-left symbol followed by *low*-left sound, *high*-right symbol followed by *low*-right sound, *low*-left symbol followed by *high*-left sound and *low*-right symbol followed by *high*-right sound. This led to 12 (3 different types of violations [P, L, PL] × 4 pairings) incongruent pairings. The number of incongruent pairs per block was randomized within 10 groups of 72 trials.

Participants were instructed how a symbol was related to a sound. They were asked to read the symbols of one row in advance and to match them with the corresponding sounds. After the onset of the response cue (“?”), participants had to evaluate the congruency of the whole trial within 2000 ms as correct as possible, see Figure 5. The left button was assigned to the left thumb after a trial with only congruent symbol-sound pairs. The right button was assigned to the right thumb after the occurrence of an incongruent pair. Each participant started with eight training blocks before preparing the EEG setup. All fulfilled the performance criteria as described in Experiment 1. Participants received a reward of 1 cent per correct response to achieve a high accuracy, in total 7.84€. Practically, participants earned between 6.91 and 7.69€ with an average of 7.32€. After each block, a visual feedback appeared about how much they earned in the block (providing the number of correct responses). Breaks were included on demand.

#### Data recording and analysis

Analog to Experiment 1, the same recording settings, pre-processing steps and parameters regarding trial exclusion, filtering, EOG correction, epoching, rejection, and balancing the numbers of events were applied. Here, the selected congruent siblings had the same physical properties regarding the pitch *and* the spatial location (and also the same position in the trial) as the corresponding incongruent sound. The epochs were averaged separately, i.e., for incongruent (P_inc, L_inc, PL_inc) and their corresponding congruent (P_con, L_con, PL_con) sounds. The incongruent-minus-congruent-difference waveforms (P = P_inc—P_con; L = L_inc—L_con; PL = PL_inc—PL_con) were computed at the grand averaged data.

All statistical tests were performed with the difference potentials. Tests in the IR range were conducted at the same bilateral ROIs as in Experiment 1 (ROI frontal: AF3-F5 and AF4-F6; ROI central: C3-C5 and C4-C6). Peak latencies were derived via jackknifing at ROI frontal for P, ROI central for L and at both ROIs for PL (cf. Experiment 1). The obtained peak latencies (PL: 109 ms at both ROIs, P: 122 ms, L: 126 ms) were tested statistically with a repeated measures ANOVA (factor violation type: PL vs. P vs. L). Due to a non-significant result [*F*_(2, 30)_ = 0.5, *p* = 0.527, ε = 0.613], a joint time window was centered at the mean of the peaks, encompassing all three peaks (104–134 ms). A repeated measures ANOVA with the factors of violation type (PL vs. P vs. L) × ROI (frontal vs. central) × hemisphere (left vs. right) tested for amplitude differences of the effects.

N2b peak latencies were determined at Fz with the jackknife method (cf. Experiment 1). The repeated measures ANOVA with the factor of violation type (PL vs. P vs. L) was followed up by paired *t*-tests of PL vs. P and L vs. P. Amplitude differences were tested with a repeated measures ANOVA (factor violation type) at Fz with separate, peak-adjusted time windows (PL: 170–210 ms, P: 185–225 ms, L: 188–228 ms). Paired *t*-tests of PL vs. P and L vs. P followed up. Voltage distribution maps were computed by using spherical spline interpolation of the scalp potential data (cf. Experiment 1).

The RTs and accuracy data were averaged separately for all congruent trials, trials with single pitch violations, with single location and with concurrent violations. Please note that RTs cannot be interpreted as participants had to wait for a response cue. A repeated measures ANOVA was conducted for the accuracy data with the factor congruency status (congruent vs. pitch violations vs. location violations vs. concurrent violations). It was followed up by two paired *t*-tests (concurrent vs. pitch violations; concurrent vs. location violations). The Greenhouse-Geisser-correction was applied when the assumption of sphericity was violated. All reported results refer to a significance level of alpha = 0.05, two-tailed.

### Results

#### Performance

**Figure 6:**
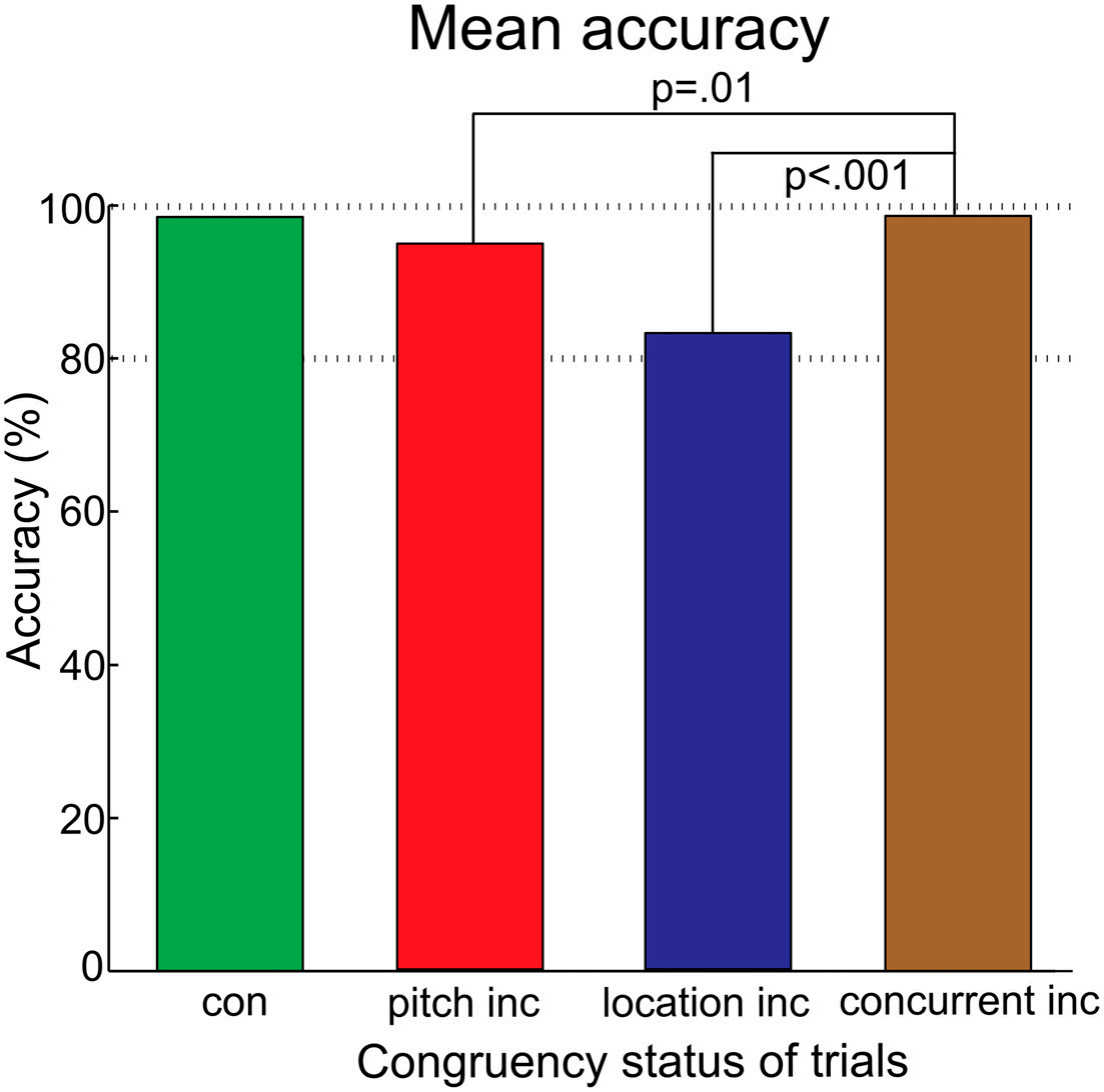
**Bar charts of the accuracy**. “Con” denotes the averaged response accuracies of all congruent trials. “Inc” (incongruent) denotes the averaged response accuracies of violation trials, separated for violation type. The percentage is higher than chance level for each violation type. This indicated that participants could solve the task. The concurrent violation yielded higher accuracies than the single violations.

A proportion of 1.3% of trials was outside the provided response window and therefore discarded. The accuracy for the congruent and the three violation types yielded higher values than chance level (50%) each. Accuracy was influenced by the congruency status [*F*_(3, 45)_ = 27.21, *p* < 0.001, η_*p*_^2^ = 0.65, ε = 0.0468]. The follow-up tests resulted in significant differences between the concurrent vs. pitch violations [*t*_(15)_ = 2.93; *p* = 0.01] and the concurrent vs. location violations [*t*_(15)_ = 5.86; *p* < 0.001], see Figure 6. [Ranking order of RTs, without statistics: Participants were fastest after congruent trials (271.51 ms), followed by concurrent violation (277.97 ms), pitch violation (278.59 ms) and location violation trials (298.60 ms)].

#### Electrophysiological components

**Figure 7:**
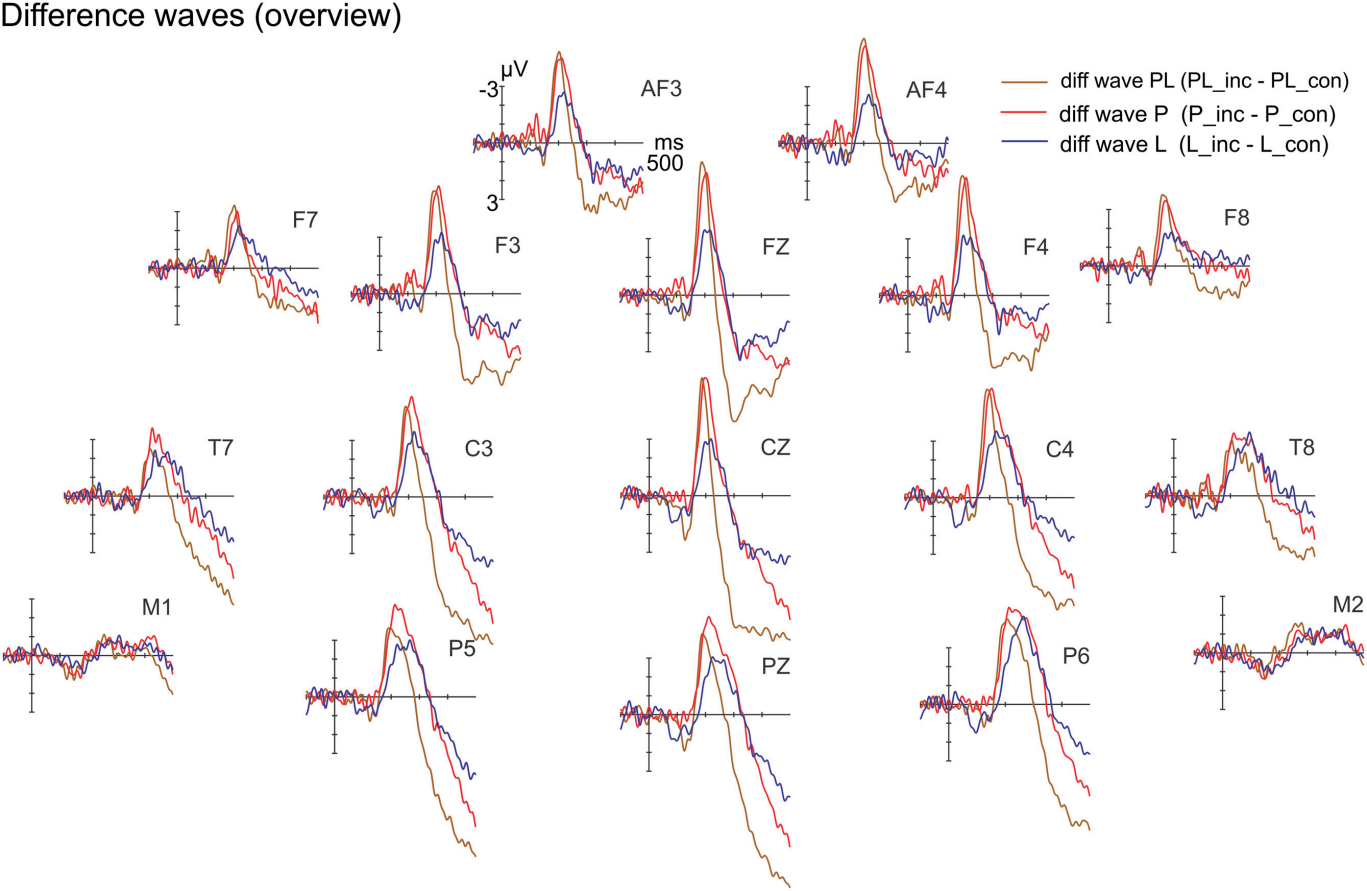
**Difference waves (incongruent-minus-congruent) for pitch (P), location (L) and concurrent violations (PL) at selected electrodes**. The expected effects in the IR time range did not yield sufficient strength.

The incongruent and congruent-sibling ERPs are provided separately in Figure S2 (Supplementary Material). Figure 7 depicts the grand average difference waveforms. The ANOVA of violation type × ROI × hemisphere on difference potentials resulted neither in significant main effects [violation type: *F*_(2, 30)_ = 1.48, *p* = 0.243; ROI: *F*_(1, 15)_ = 3.49, *p* = 0.082; hemisphere: *F*_(1, 15)_ = 3.57, *p* = 0.078] nor interactions [violation type × ROI: *F*_(2, 30)_ = 1.59, *p* = 0.222; violation type × hemisphere: *F*_(2, 30)_ = 1.19, *p* = 0.320; ROI × hemisphere: *F*_(1, 15)_ = 1.1, *p* = 0.320; violation type × ROI × hemisphere: *F*_(2, 30)_ = 0.3, *p* = 0.745]. The constant term (intercept) expresses the impact of the congruency averaged over all conditions in the difference potential. It was n.s. [*F*_(1, 15)_ = 0.01, *p* = 0.945]. Due to the absence of one factor of the additive model, it could not be tested in a meaningful manner.

(A variety of other repeated measures ANOVAs at IR range resulted in also n.s. effects. For instance, smaller, separate peak-adjusted time windows at the obtained latencies (PL: 99–119 ms, P: 112–132 ms, L: 116–136 ms) were used to maximize IR effects. Additionally, the IR peak latencies of P and L from Experiment 1 were tested together with a jackknifed peak latency for PL from the present data. Only a partial repeated measures ANOVA of violation (PL vs. P) × hemisphere (left vs. right) at ROI frontal showed any effect. The intercept was significant [*F*_(1, 15)_ = 8.56, *p* = 0.01]. However, this model is not fitting to the design as it is partly excluding the location manipulation).

**Figure 8:**
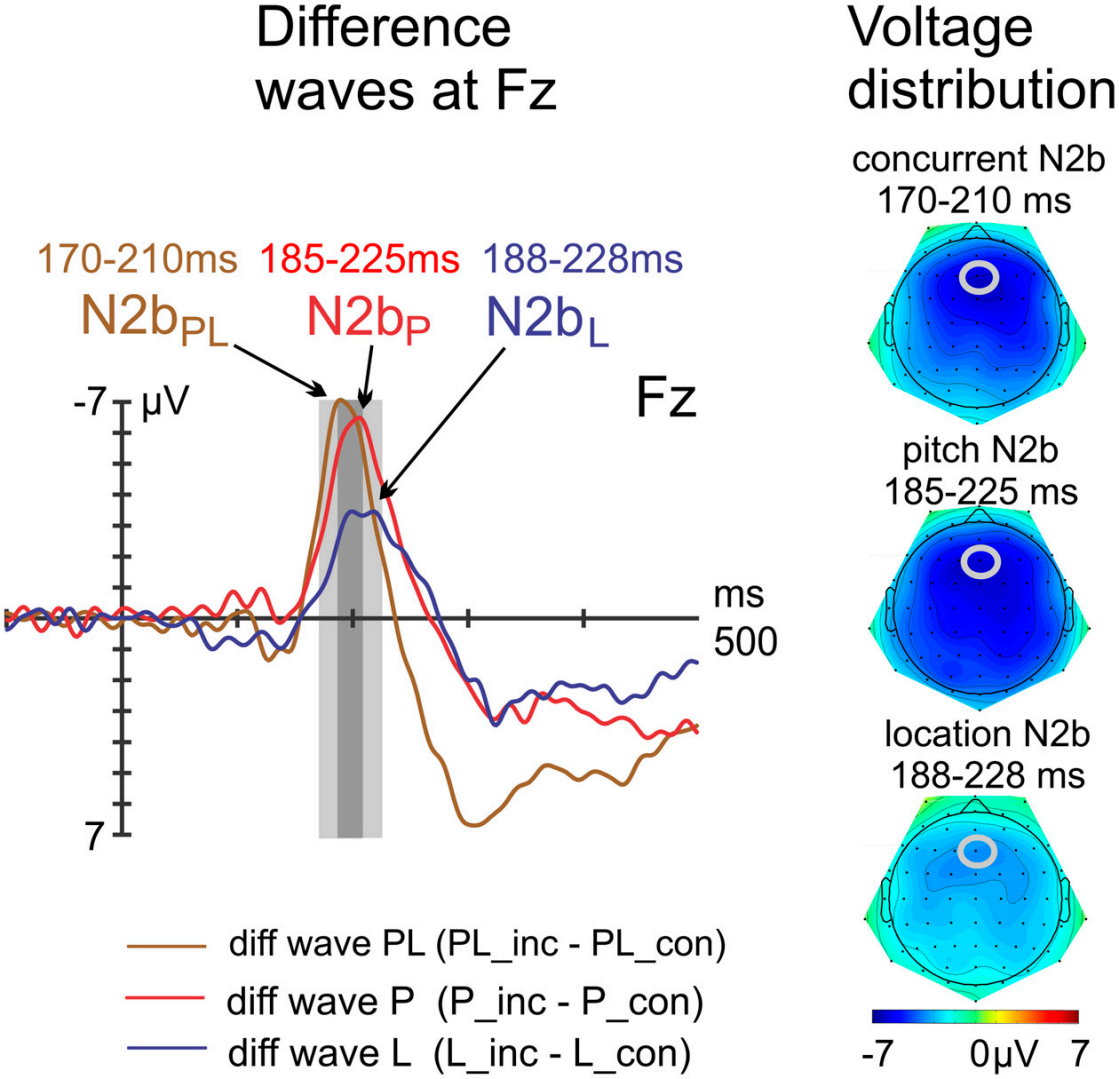
**Difference waves at electrode Fz, including voltage distribution maps of N2b**. The N2b peaked earlier in response to concurrent violations but was of similar amplitude compared with the N2b in response to pitch violations.

The N2b peak latencies (PL: 190 ms, P: 205 ms, L: 208 ms—i.e., the second of the double peak was determined via jackknifing) were significantly different [main effect of violation type: *F*_(2, 30)_ = 11.25, *p* = 0.004, η_*p*_^2^ = 0.43, ε = 0.509]. Both follow-up *t*-tests resulted in significant differences, i.e., concurrent vs. pitch violations [PL vs. P: *t*_(15)_ = −3.28, *p* = 0.005] and pitch vs. location violations [P vs. L: *t*_(15)_ = −3.55, *p* = 0.003]. Amplitude differences with peak-adjusted time windows (PL: 170–210 ms, P: 185–225 ms, L: 188–228 ms) resulted in the main effect of violation type [*F*_(2, 30)_ = 9.06, *p* < 0.001, η_*p*_^2^ = 0.38]. Follow-up *t*-tests tests resulted in a non-significant comparison of PL vs. P [*t*_(15)_ = −0.28, *p* = 0.786] and a significant comparison of P vs. L [*t*_(15)_ = −3.68, *p* = 0.002]. The constant term (intercept) which reports the effect of congruency averaged over all conditions was highly significant [*F*_(1, 15)_ = 48.29, *p* < 0.001, η_*p*_^2^ = 0.76]. The topographical maps of the N2b components show a fronto-central voltage distribution as expected (Figure 8).

### Discussion

Experiment 2 aimed to investigate the processing of sounds at sensory and cognitive-attentive levels when a sound violated two predictions concurrently. In the paradigm, each symbol predicted two feature values of the upcoming sound. The prediction was rarely violated either in terms of pitch, location or in both features concurrently. Participants had to evaluate the congruency of the whole trial. The results confirmed our hypotheses only partly. The accuracies of task performance indicated that all violation types were discovered. While the IR did not reach significance, N2b components were clearly obtained for each violation type. Additionally, concurrent violations were processed with temporal advantage when compared with the single violations. In the following, the results are associated with each other and explained in detail.

The accuracy data confirm that the demanding task could be solved at the highest abstraction level. While location violations were hardest to detect of all, concurrent violations were better detected than either of the single violations. The latter result speaks in favor of a parallel, i.e., concurrent processing of the two features. However, the comparison of the inputs may have been performed retrospectively. The focus at the previous processing levels could reveal the specifics of the processing with regard to predictive mechanisms.

Considering the results of Experiment 1, we assumed that each violation type would elicit the IR. To control a priori for an appropriate ratio of significance level (α = 0.05) and power of the test (commonly accepted: 1-ß = 0.8), the required sample size was calculated. (The sample size was estimated with the G-Power 3.1.9 software, available at www.gpower.hhu.de; Faul et al., 2007. The analysis was based on the Three-Way interaction from the repeated measures ANOVA on IR amplitudes of Experiment 1. The obtained effect size of η_*p*_^2^ = 0.59 was transformed into Cohen's effect size as recommended in the tool). Eleven participants would suffice to separate random from significant effects. 16 participants were included to parallelize as much as possible with Experiment 1. As power should have been sufficient to detect a significant IR, we dare to speculate why—contrary to our expectations—the best fitting ANOVA did not yield any significant IR effects. Firstly, the absence of the IR may indicate that the sensory matching process did indeed not occur. The sensory prediction might be represented only with a low signal-to-noise ratio. Attentional capacity limitations, i.e., a bottleneck, might have occurred due to the fast stimulation or the concurrent processing. The task was demanding as it required focusing on two modalities and two features in a short time to intentionally predict the sound. The auditory system might not have been able to detect a prediction violation reliably in time or in a prospective manner. This finding would differ from findings of MMN studies (Levänen et al., 1993; Schröger, 1995): MMN of both features was not affected by concurrent processes. Secondly, there might be other reasons why the IR signal was too weak. If an expected component cannot be measured or fails statistical significance, it does not necessarily mean that the underlying process did not occur (Schröger, 2007). Due to the loss of false-response trials, the signal-to-noise ratio in the IR measurement might have been too low. This seems especially valid in response to the location violations, as they showed the lowest accuracy and therefore the highest loss of data. Remarkably, the statistically significant intercept of the partial ANOVA, ignoring the single location violations, suggests tentatively that at least the pitch violations were detected at sensory levels. However, the sensory processing of the sound and the subsequent categorization were presumably not impaired, as the N2b range reveals (see below).

Subsequently, N2b was elicited by all violation types. In attended auditory oddball paradigms, N2b is assumed to reflect the detection of targets at the higher cognitive-attentive level (Ritter et al., 1979; Novak et al., 1990). This level involves categorial representations (Näätänen and Winkler, 1999). In the present experiment, the rare violations were presumably selected as targets as their identification and categorization was necessary to fulfill the task (cf. Widmann et al., 2007, 2012). So N2b presumably indexes the attentive detection of a prediction violation (Lindström et al., 2012) mainly on the basis of categorial representations. The short latency of N2b indicates that a retrospective comparison is unlikely. Rather, prospective processing occurred.

Assuming that the sensory matching processes underlying the IR did not occur, the serial model of the detection of regularity violations would be contradicted. In this model, the N2b is regarded as the forwarded error signal of a pre-attentive sensory error signal, as indexed by MMN (Näätänen et al., 1982; Novak et al., 1990; Tiitinen et al., 1994; Horváth et al., 2008). Sensory predictions based on auditory input are derived within the modality in the auditory cortices. In the present paradigm, the regularities were presumably processed at higher cognitive levels. The abstract representations of the predictions were presumably conveyed “down” the hierarchy. At each level, the predictions are likely to be transformed into appropriate representations (cf. Mumford, 1992). Presumably, in the present experiment they were not conveyed as far as to the auditory system as proposed by Widmann et al. (2007). We conclude that the N2b elicitation may not (only) depend on an early auditory-sensory detection mechanism. Rather, the findings may suggest an independent detection mechanism at a hierarchically higher level. The predictions need not necessarily to be represented and matched at sensory levels. Categorial (abstract) predictions may serve at higher levels for the same purpose and may suffice. Thus, the processing paths between MMN and IR may differ.

However, amplitudes of the single N2b components differed in the present experiment, whereas they were comparable in Experiment 1. In the present study, the reduced N2b amplitude in response to location violations might reflect a lacking contribution from sensory levels (which may have been present for pitch violations). Hence, the present findings may also support the serial processing model with respect to the amplitude differences. Alternatively, in some location trials a later, retrospective comparison might have occurred which cannot be reflected at the N2b level. Concurrent violations did not have N2b of higher amplitude compared with the single pitch violations. The high N2b amplitude of pitch violations suggests that these single violations already exhausted the neural capacities, leading to a ceiling effect.

Further, concurrent violations showed a latency shortening of N2b compared with both single violations. This suggests a parallel, i.e., concurrent processing of pitch and location violations (as already suggested by the accuracy data). The present data set does not allow any conclusion as to whether the categorial prediction violations were processed in parallel as proposed by the separate activation account (“race models”) or in parallel as proposed by the interactive activation account (cf. Miller, 1991). We speculate that the latency shortening may indicate the higher salience of concurrent violations due to an interaction of both pieces of information (cf. Schröger, 1995; Pieszek et al., 2013).

Altogether, the alternative hypothesis can be only partly rejected. Presumably, the demanding task had an impact on early sensory processing (no significant IR) and the task performance (not equal for all violation types). The present data could not reveal whether and how two intentional symbolic predictions are maintained and tested already at the sensory level. The lack of significant IR effects at the sensory level prevented the meaningful application of the additive model (PL = P + L) to test for processing specifics (cf. Vroomen and Stekelenburg, 2010; Pieszek et al., 2013). As the sample size exceeded the a priori determined necessary sample size, the non-significant results may very likely reflect random processes. We speculate that the signal-to-noise ratio of the representation of the sensory prediction might have been too low. Finally, symbolic predictions seemed to not reliably feed backward to and/or maintained at sensory levels of processing. In future studies, simplifying the task (e.g., by defining a longer SOA) might already suffice to observe effects in the IR range. Additionally, an eye tracker would improve the design to check the real predictive interval for each symbol-sound-pair. Aoyama et al. (2006) found that 50 ms are not long enough, whereas 300 ms suffice to establish a sound prediction. A selection of trials regarding this aspect may also yield a higher signal-to-noise ratio to reveal the expected IR.

However, the brain was able to establish concurrent intentional predictions and detect violations at latest at cognitive-attentive levels. This was reflected by N2b and high accuracies in response to all violation types. Especially, the processing advantage for the concurrent violations at the cognitive and behavioral level indicated that predictions were successfully established for both sound features. Näätänen and Winkler (1999) suggested that the processing of the information might only be postponed to a later level if earlier components were not observed. Corroborating this assumptions and our findings, Lindström et al. (2012) and Widmann et al. (2012) did not obtain effects in the sensory range but N2b in a similar context. We suggest that there might be an independent matching mechanism besides triggering the N2b by forwarded sensory prediction errors.

## General discussion and conclusion

In Symbol-to-Sound matching paradigms, rare violations of intentional predictions occurred. The participants' task was to indicate congruent and incongruent trials. Violations of such predictions can be processed at different hierarchical levels. At a modality-specific, early level, the elicitation of IR presumably reflects a sensory prediction error. It is presumably generated by a mismatch of input and automatically pre-activated auditory sensory representations (Widmann et al., 2007, 2012; cf. Zatorre and Halpern, 2005). At the later cognitive-attentive level, the elicitation of N2b reflects a higher-order prediction error. Experiment 1 and 2 differed regarding the expected sensory prediction error. The IRs could be observed in Experiment 1, but not in Experiment 2. In both experiments, N2b components were obtained. This indicates that the attentive detection of the prediction violation was signaled for both sound features.

The hierarchical principle as proposed by the predictive coding theory (e.g., Friston and Kiebel, 2009) may explain the divergent results. Theoretically, the symbolic predictions generated in higher-order areas are conveyed to lower cortical areas (cf. Widmann et al., 2007). The descending prediction representations are presumably matched with ascending information at different levels (cf. Clark, 2013). Experiment 1 showed that a sensory prediction that originated from higher areas was conveyed as low as to a feature-specific sensory area. These results support the functional model of symbolic prediction (Widmann et al., 2007). Moreover, feature-specific mismatch signals revealed the symbolic predictive process for different sound features. Hence, predictive models at sensory levels seem to be distributed in a feature-specific manner. Violations were also indexed at the higher, cognitive-attentive level. We conclude that predictions were derived, maintained and matched with the sound for the pitch and for the location feature. In Experiment 2, the task load and complexity of the design increased. Two sound features had to be predicted concurrently and at fast pace. It appeared that matching was not reliably implemented at the sensory level, as the sensory prediction error was less evident. The top-down information was presumably not (fully) available due to capacity limitations in the backward propagation of the prediction. This may have resulted in low signal-to-noise ratio of the sensory representation of the prediction. However, the pitch and location information were presumably adequately processed and categorized at a higher level (cf. Näätänen and Winkler, 1999). The successful attentive violation detection suggested a hierarchical, partly independent encoding of information for both features. Remarkably, there was an advantage in processing for the concurrent violations. Thus, symbolic predictions seem to be maintained and tested concurrently latest at cognitive-attentive levels. Moreover, the task might be solved mainly on basis of categorial, i.e., abstract information (whereby sensory representations presumably co-exist, Näätänen and Winkler, 1999). The higher-order N2b indexes the violation of a visual-auditory regularity in a predictive situation more reliably than the IR.

### Conflict of interest statement

The authors declare that the research was conducted in the absence of any commercial or financial relationships that could be construed as a potential conflict of interest.

## Supplementary material

Figure S1 **Auditory ERPs and difference waves of the pitch (A) and the location (B) condition in Experiment 1**. For each dataset, a corresponding congruent sound (“sibling”; P_con, L_con) was randomly selected for each incongruent sound (P_inc, L_inc). The difference potentials (diff wave P and L, in black) were computed as incongruent-minus-congruent ERPs at each time point. The difference waves reveal the effects of violations of symbolic predictions for each sound feature.

Figure S2 **Auditory ERPs and difference waves of the concurrent violations (A), the pitch (B) and location violations (C) in Experiment 2**. Difference waves were calculated as incongruent-minus-congruent sibling sounds for each violation, analog to Experiment 1.
