# Supplementary figures and images for: Separate and concurrent symbolic predictions of sound features are processed differently
Source: Front Psychol. 2014 Nov 18;5:1295. doi: 10.3389/fpsyg.2014.01295 (PMC4235414; doi:10.3389/fpsyg.2014.01295)

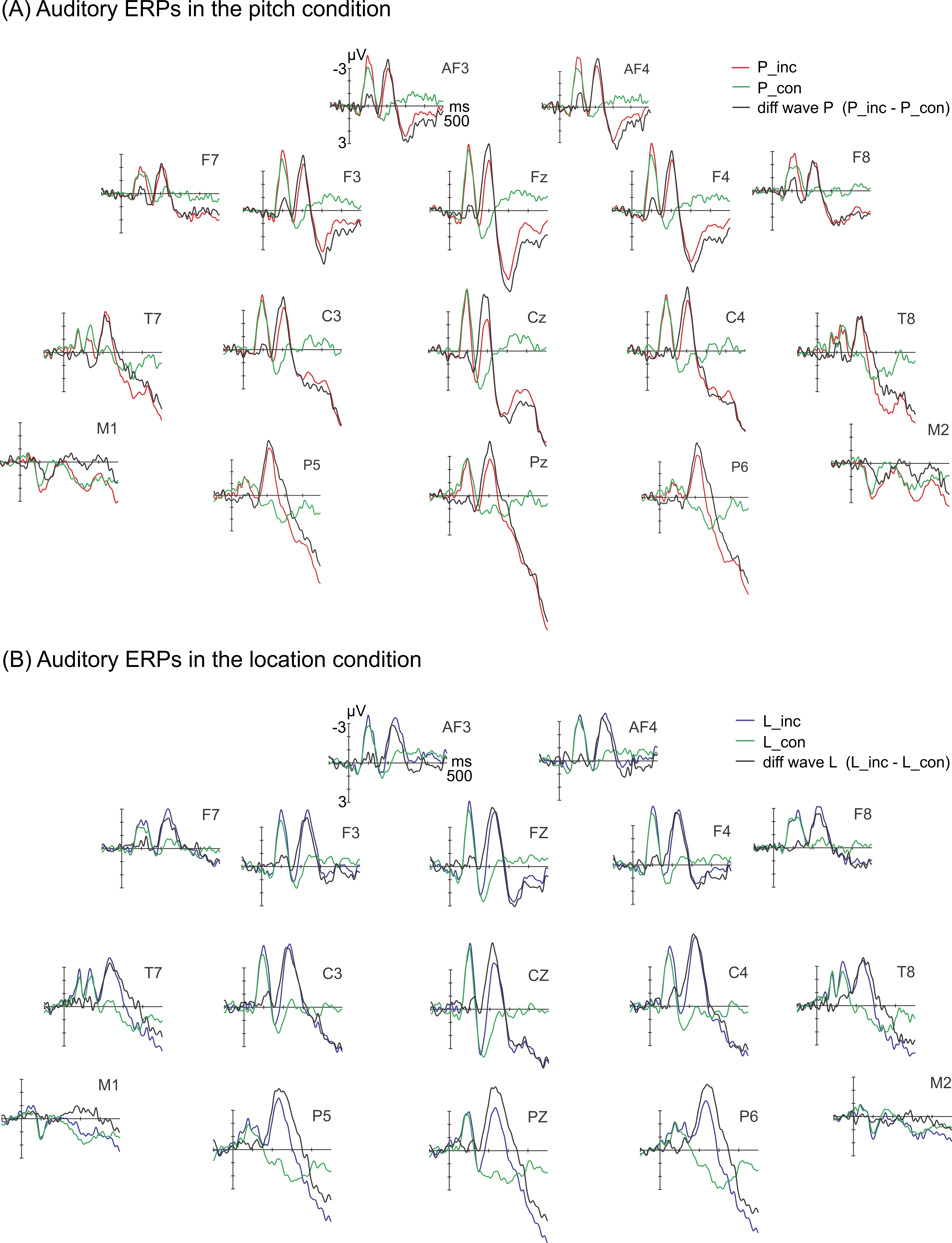

Supplement: Figure S1 — Auditory ERPs and difference waves of the pitch (A) and the location (B) condition in Experiment 1. For each dataset, a corresponding congruent sound (“sibling”; P_con, L_con) was randomly selected for each incongruent sound (P_inc, L_inc). The difference potentials (diff wave P and L, in black) were computed as incongruent-minus-congruent ERPs at each time point. The difference waves reveal the effects of violations of symbolic predictions for each sound feature. [file FigureS1.TIF]
